# Cloning of Maize *TED* Transposon into *Escherichia coli* Reveals the Polychromatic Sequence Landscape of Refractorily Propagated Plasmids

**DOI:** 10.3390/ijms231911993

**Published:** 2022-10-09

**Authors:** Chunsheng Cong, Jingsheng Tan, Chuxi Li, Fangyuan Liu, Qian Yu, Li Zhu, Yubin Li

**Affiliations:** 1 Biotechnology Research Institute, Chinese Academy of Agricultural Sciences, Beijing 100081, China; 2 College of Agronomy, Qingdao Agricultural University, Qingdao 266109, China

**Keywords:** maize, *Mutator* superfamily, *TED*, *Jittery*, cloning, *Escherichia coli*, plasmid

## Abstract

*MuDR*, the founder member of the *Mutator* superfamily and its MURA transcripts, has been identified as toxic sequences to *Escherichia coli* (*E. coli*), which heavily hindered the elucidation of the biochemical features of MURA transposase and confined the broader application of the *Mutator* system in other organisms. To harness less constrained systems as alternatives, we attempted to clone *TED* and *Jittery,* two recently isolated autonomous *Mutator*-like elements (*MULEs*) from maize, respectively. Their full-length transcripts and genomic copies are successfully cloned when the incubation time for bacteria to recover from heat shock is extended appropriately prior to plating. However, during their proliferation in *E. coli*, *TED* transformed plasmids are unstable, as evidenced by derivatives from which frameshift, deletion mutations, or IS transposon insertions are readily detected. Our results suggest that neither leaky expression of the transposase nor the presence of terminal inverse repeats (TIRs) are responsible for the cloning barriers, which were once ascribed to the presence of the Shine–Dalgarno-like sequence. Instead, the internal sequence of *TED* (from 1250 to 2845 bp), especially the exons in this region, was the most likely causer. The findings provide novel insights into the property and function of the *Mutator* superfamily and shed light on the dissection of toxic effects on cloning from *MULEs*.

## 1. Introduction

Transposable elements (TEs) or transposons are DNA segments that can change locations or multiply within host genomes via transposition [1]. TEs can be classified as Class I TEs (retrotransposons) or Class II TEs (DNA transposons) on the basis of their structural and biochemical features [2]. The *Mutator* superfamily can make up the majority of Class II or DNA TEs in plant genomes [3,4,5,6]. The autonomous or master element of this superfamily is *MuDR* [7]. Numerous transposons similar to *MuDR* have been found in a number of sequenced genomes including plants [8,9,10,11,12], fungi [13,14], protozoa [15,16], and multicellular animals [17,18,19]. Due to their similarity to *MuDR*, these elements are also known as *Mutator*-like elements (*MULEs*). At present, most of the identified *MULEs* are non-autonomous transposons that cannot encode fully functional transposases by themselves. Only a few *MULEs* can transpose autonomously such as *Hop* in *Fusarium oxysporum* [14], *AtMu1* in *Arabidopsis thaliana* [20], *Jittery* [21] and *TED* [22] in maize, *Os3378* in rice (*Oryza sativa*) [23], and *Muta1* in mosquito (*Aedes aegypti*) [24]. However, knowledge of *MULEs* transposition and regulation in maize traces entirely to genetic studies of three autonomous elements, *MuDR* [7,25,26,27,28,29,30,31], *Jittery* [21], and *TED* [22]. Interestingly, neither the maize autonomous *MuDR* nor *MuDR*-like elements from the Mexican land race Zapalote chico (*MuDR-Zc*) can be maintained stably in *E. coli*, even in recombination defective strains [26,32,33]. The *mudrA* cDNA is also extremely difficult to propagate in *E. coli*, as cloning products often bear frame-shift mutations in MURA due to point insertions or deletions [34]. The Shine–Dalgarno motif, in front of prokaryotic start codons, could guide the translation of intracellular mRNAs [35], which is proposed to result in the instability of *MuDR* from allowing the production of transposase in *E. coli* [36].

The *MuDR/Mu* system, with its dual advantage of high transposition frequency and genic region targeting, has provided thousands of mutant lines for maize forward and reverse genetics [37,38,39]. However, the use of *Mu* elements may be short of achieving genome saturation, largely because of the insertion preferences evident from the sequence analysis of independently constructed *MuDR/Mu* insertional libraries [39]. In addition, the application of the *MuDR/Mu* system to the production of plant mutant collections is still confined to maize in contrast to the maize *Ac*/*Ds* system, which has been deployed in several plant species [40,41,42,43,44,45,46].

The discovery of two other autonomous *MULEs*, *Jittery* and *TED*, prompted us to characterize their structure, genetic behavior, and transposase biochemistry in order to obtain a more global picture of the *Mutator* superfamily. Their characterization may also enable us to construct new mutant libraries to complement those obtained with *MuDR/Mu*. *TED* was identified as a *MULE* insertion in the *bz* second exon of the *bz-m175* mutable allele arisen in a High Loss/High Knob maize stock. It produces a fine-spotted phenotype as a result of late transposon excisions during aleurone development [22]. *Jittery* was also identified as a *MULE* insertion in the *bz* second exon of a mutable allele, *bz-m039*. This mutant, which arose in a maize stock infected with the barley stripe mosaic virus (BSMV), produces a heavily, fine-spotted seed phenotype, also from late transposon excisions during aleurone development [21]. There are some commonalities between these two transposons and *MuDR*. For example, both have sequences homologous to *mudrA*, long terminal inverted repeats (TIRs) of a 200-bp length, and the 9-bp target site duplications (TSDs) of flanking sites. However, *Jittery* and *TED* share some common characteristics that distinguish them from the *MuDR/Mu* system such as low copy number, high reversion frequency in germ cells, and the absence of *mudrB* homologous sequences [47].

In this study, we clarified the gene structure and transposase characteristics of both *TED* and *Jittery*, two important *MULEs* in maize. Furthermore, we found that, similar to *MuDR*, both the full-length genomic DNA and cDNA of *TED* are refractory to be cloned in *E. coli.* Serendipitously, we modified the standard protocol of cloning and cloned the *TED*-related sequences, which allowed us to study the dynamics of plasmid DNA replication and to dissect the hypothetic toxic sequence structures or compositions impairing the normal growth of bacteria. Our findings shed light on the *Mutator* superfamily of transposons in maize and provide a basis for the study of the toxic effects on cloning from *MULEs*.

## 2. Results

### 2.1. Gene Structure and Transposase Characteristics of TED and Jittery

**Figure 1:**
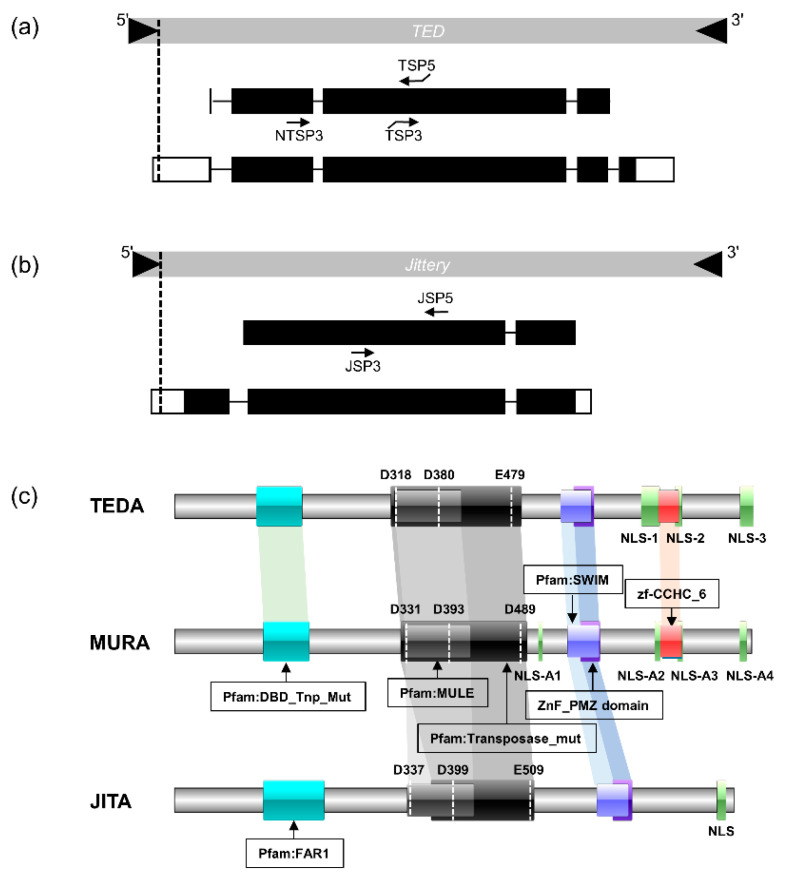
Schematic representation of the gene structure, transcript, and transposase. (**a**) Diagram of the *TED* gene structure and its transcript, showing comparison of the predicted TEDA coding and noncoding sequences (GenBank: KF287636) with the cloned TEDA transcript (GenBank: ON497071). *TED* TIRs are triangles, open boxes are 5′-UTR and 3′-UTR, closed dark boxes are exons, and lines in between boxes are introns. The arrows show the primers used in RACE (primer sequences are in Supplementary Materials Table S1). The vertical dashed line marks the 3′-end of *TED* 5′-TIR. (**b**) *Jittery* transposon and its gene structure. The top part is the *Jittery* genomic structure and the black triangles represent the TIRs. The middle part is the predicted CDS (GenBank: AF247646) and the bottom part is the full-length cDNA cloned via RACE (GenBank: ON497072). Open boxes are 5′-UTR and 3′-UTR, closed dark boxes are exons, and lines in between boxes are introns. The arrows show the primers used in RACE (primer sequences are in Supplementary Materials Table S1). The vertical dashed line marks the 3′-end of *Jittery* 5′-TIR. (**c**) Domain conservation among TEDA, MURA (GenBank: AAA81535), and JITA. Colored boxes are the different conserved domains. White vertical dotted lines are the DDE triad conserved among the *Mutator* transposases (Supplementary Materials Figure S1). Light green boxes are the conserved nuclear localization sequence (NLS). Shaded regions are in common among these transposases.

*TED* and *Jittery* are two important *MULEs* in maize. They are plausible avenues of research, suggested by their similarities and uniqueness of the gene structure and transposase characteristic with *MuDR,* on the further understanding and wide application of the *Mutator* superfamily in plants. To characterize the major transcripts encoded by these two elements, we cloned the full-length cDNA of *TED* and *Jittery* by means of the rapid amplification of the cDNA ends (RACE), respectively. The *TED* full length cDNA sequence was 3098 bp including a 379-bp 5′-untranslated region (UTR) initiating from the 3′-end of the 5′-TIR and a 241-bp 3′-UTR ending with a poly (A) tail (Figure 1a, GenBank: ON497071). Compared to the predicted CDS (GenBank: KF287636) [22], it was extended to four introns and five exons by an additional splicing site. The last splicing site was upstream of the theoretical stop codon and led to a novel exon where the exon–intron junctions conformed to the GT-AG rule (Figure 1a). The *Jittery* full-length cDNA measured 2738 bp in length and was composed of three exons with a 230-bp 5′-UTR and a 111-bp 3′-UTR. The 5′-UTR sequence of *Jittery* cDNA extended into the 3′-end of the 5′-TIR sequence in line with the *TED* 5′-UTR (Figure 1b, GenBank: ON497072). Compared to the earlier predicted CDS (GenBank: AF247646) [21], there was an extra splice site upstream of the theoretical exon 1, leading to a novel exon and intron conforming to the GT-AG rule at the splicing site.

The cloned *TED* full-length cDNA encoded a 94-kD transposase, TEDA, of 825 amino acid residues and was homologous (47.6% identity and 62.4% similarity) to MURA (Figure 1c top) while *Jittery* was predicted to encode a transposase of 798 amino acid residues with a molecular weight of 91 kD and only had a distant homology (18.0% identity and 28.8% similarity) to MURA (Figure 1c bottom). The domain conservation analysis revealed that both TEDA and JITA shared some conserved domains with MURA such as Pfam: MULE, Transposase_mut, SWIM, and ZnF_PMZ_domain (Figure 1c). Compared with the transposase JITA, TEDA and MURA showed more conserved protein domains including Pfam: DBD_Tnp_Mut and zf-CCHC_6. At the N-terminus of transposase JITA, there was a FAR1 DNA-binding domain, which shares a stable core with DBD_Tnp_Mut, making both of them members of the WRKY-GCM1 protein superfamily. The multiple alignment of transposases from the *Mutator* superfamily showed that both TEDA and JITA contained the conserved DDE motif (TEDA: D318, D380 and E479; JITA: D337, D399 and E509) (Figure 1c and Supplementary Materials Figure S1). The predicted subcellular localization of TEDA is in the eukaryotic nucleus, where transposition occurs. Analysis of nuclear localization sequences (NLS) revealed three putative NLSs at the C-terminal of TEDA (Figure 1c, top and Supplementary Materials Table S2). Among them, NLS-1 is located in exon 3, NLS-2 is shared by exon 3 and exon 4, and NLS-3 is located in exon 5, the last exon. Sequence alignment results indicated that these three nuclear localization signals predicted in TEDA aligned with three functional NLSs in MURA that have been validated in vitro, respectively [48] (Supplementary Materials Table S2), supporting the completeness of the cloned transcript of TEDA. JITA was also predicted to be located in the eukaryotic nucleus, but only one NLS was predicted at the C-terminal end (Figure 1c and Supplementary Materials Table S2), which was different from MURA and TEDA (Figure 1c).

### 2.2. TED Full-Length Genomic DNA and cDNA Are Refractory to Be Cloned in E. coli

**Figure 2:**
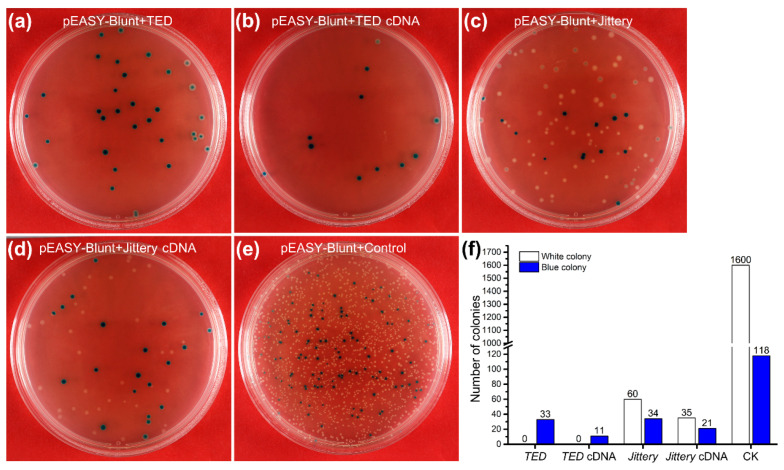
Growth dynamics of the colonies transformed with different insertional sequences by the standard cloning method. The simultaneous studies show that *Jittery* and its full-length cDNA are less toxic to *E. coli* in contrast to *TED* and its full-length cDNA, in spite of much lower numbers of colonies, either white or blue, than the control sample. Pictures of the plating culture were taken at 24 h of incubation at 37 °C. (**a**) *TED* genomic DNA (3960 bp). (**b**) *TED* full-length cDNA (3098 bp). (**c**) *Jittery* genomic DNA (3914 bp). (**d**) *Jittery* full-length cDNA (2738 bp). (**e**) Positive control from the cloning kit (700 bp). (**f**) The counts of different colonies from the corresponding plating culture of different cloning inserts (**a**–**e**).

The difficulty of cloning *MuDR*-related sequences in *E. coli* remains a major obstacle toward a more complete biochemical analysis of the MURA transposase and the practical application of *MULE*s as transposon tags in heterologous hosts. Here, we attempted to clone *TED*, *Jittery*, and their full-length cDNAs in *E. coli*, but these sequences showed varying degrees of difficulty when cloned through standard procedures. With conventional experimental procedures, *TED* and its cDNA could not be cloned in *E. coli* since there were no visible positive colonies on the growth medium (Figure 2a,b,f). *Jittery* and its cDNA could be cloned because there were some positive colonies, but the number of colonies was much fewer than that of the control provided with the cloning kit (Figure 2c–f). The full-length genomic sequence of *Jittery* was surprisingly easier to clone than its cDNA, suggesting that the *Jittery* cDNA sequence is more toxic than its genomic copy to the *E. coli* expression system. Therefore, the refractoriness to cloning in *E. coli* is not unique to *MuDR*-related sequences, an observation that supports the prior phylogenetic distance analysis of these three transposases [22,49]. In addition, these results also indicate that the sequences in the *TED* exons might be the causes of the toxic effect, since either the full-length genomic DNA or cDNA of *TED* was refractory to be cloned.

### 2.3. TED and Its cDNA Are Cloned by Appropriately Extending Bacterial Recovery Time Prior to Plating

We carried out a number of trials under different experimental conditions including tests of different ligation methods (blunt end cloning, restriction digestion and religation, and In-Fusion cloning), transforming various strains of *E. coli* competent cells (*Trans*1-T1 from TransGen Biotech, DH5α from GenStar, and Trelief ™ 5α from Tsingke Biotechnology Co), incubating the plating culture at a lower temperature, or plating with IPTG and the X-Gal free LB medium. All attempts failed to yield any transformed colonies of *TED* and its cDNA from at least two technical replicates (Figure 2a,b). Although some white colonies occasionally appeared on the medium, none of them turned out to be insertional transformants upon subsequent molecular characterization. The results elucidate that the cloning barrier seems to be irrelevant to the ligation methods, competent cell strains, or incubation temperature for plating culture.

**Figure 3:**
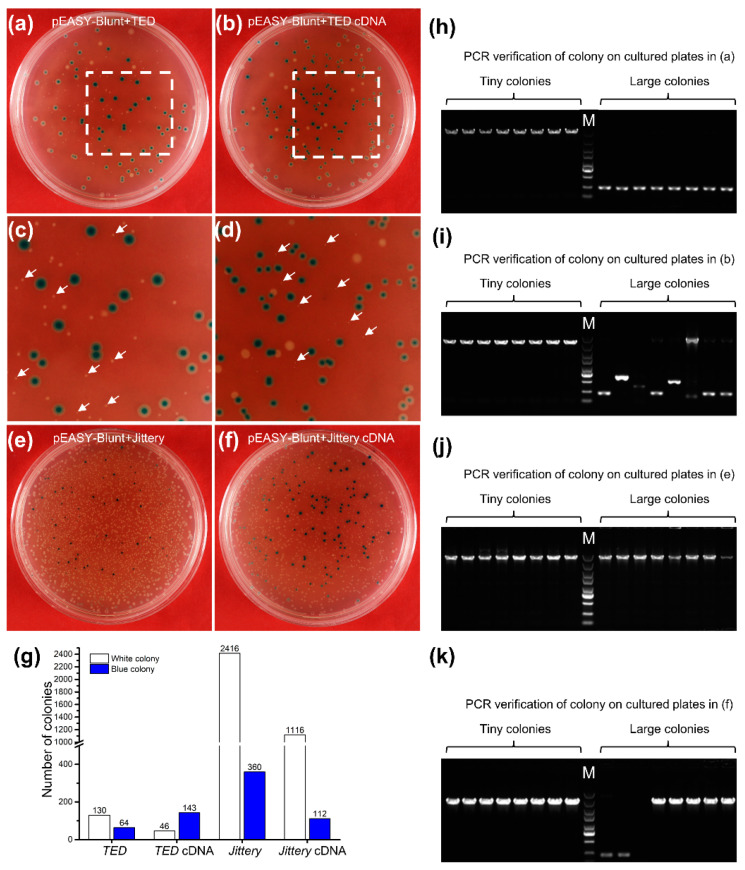
Growth dynamics of the colonies transformed with different insertional sequences by the modified cloning procedure and PCR verification of different transformed colonies. By extending the recovery time of transformed *E. coli* in LB broth, transformants with cloned *TED* and its full-length cDNA appeared as white colonies with much smaller sizes in comparison with the blue colonies, in spite of much lower numbers, either white or blue, than the *Jittery* and its full-length cDNA transformants. The plating culture growth of PCR amplicons was examined after 36 h of incubation at 37 °C. Colony PCR verification and subsequent sequencing analysis showed that positive clones bearing intact inserts were from colonies growing retardedly (Figure 2). PCR primer combination, M13F/M13R. (**a**) *TED* genomic DNA, (**b**) *TED* full-length cDNA, (**c**) A partial enlarged view of (**a**), (**d**) A partial enlarged view of (**b**). Arrows indicate tiny colonies. (**e**) *Jittery*, (**f**) *Jittery* full-length cDNA, (**g**) The counts of different colonies from the cultures of different cloning inserts (**a**,**b**,**e**,**f**). (**h**–**k**) Colony PCR verification of tiny white colonies (to the left of DNA ladder in the middle) and large colonies (to the right of DNA ladder) on cultured plates in (**a**,**b**,**e**,**f**), respectively. M, DNA ladder of 5 kb, 3 kb, 2 kb, 1.5 kb, 1 kb, 750 bp, 500 bp, 250 bp, and 100 bp.

We transformed the *Trans*1-T1 competent cells and incubated the culture in a rotary shaker at 200 rpm at 37 °C, which allows the bacteria to recover and express the antibiotic resistance gene carried by the plasmid. When we serendipitously extended the incubation time from one-hour, as recommended in the manufacturer’s protocol, to 70 min, without altering any other steps or parameters in the protocol, we were able to cultivate some tiny colonies bearing *TED* and its cDNA transformants on the LB agar plates (Figure 3a–d,g), as revealed by the subsequent molecular characterization of all positive colonies (Figure 3h,i). Furthermore, the number of tiny positive colonies was significantly lower and the growth of the positive bacterial colonies was greatly retarded in comparison to that of the control. By extending the recovery time, many more colonies of *Jittery* and *Jittery* cDNA transformants appeared on the LB medium with no retarded growth of the variably-sized colonies (Figure 3e–g), the majority of which turned out to be positive transformants (Figure 3k). Moreover, the amount of plasmid DNA extracted with the standard mini-preparation from the *TED* and *TED* cDNA positive colonies was extremely limited, which may also result from the retarded growth of the bacterial culture. Taken together, the colonies transformed with the *TED* or *TED* cDNA sequences can only survive with sufficient recovery time and have a retarded growth rate either on LB agar plates or in LB broth.

### 2.4. TED Is Highly Unstable and Hard to Maintain Intact within Bacterial Plasmids

**Figure 4:**
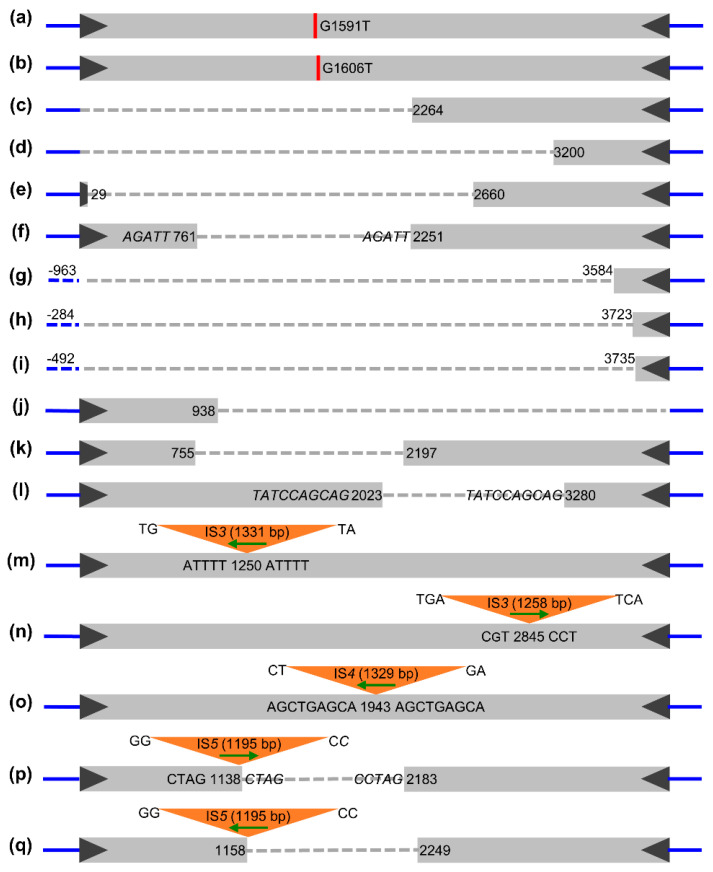
Sequence characterization of de novo mutations from *TED* transformants. (**a**,**b**) Point mutations, (**c**–**l**) Solo-terminus deletions or internal deletions (**m**–**q**) Insertions of IS transposon from the *E. coli* host genome. The gray rectangles are the *TED* nucleic acid sequence ended with the black triangles of *TED* TIRs flanked by the blue lines of the plasmid vector sequences. Deletions are shown as dotted gray or blue lines, the orange triangles are insertions of IS transposons with varied sizes (in parenthesis), and the horizontal green arrows indicate the orientation of IS elements. Insertion sites are labeled with numbers flanked by TSD sequences, and the italics represents the micro-homologous sequence.

Previous studies have indicated that both the *MuDR* and *MuDR-Zc* elements were difficult to maintain in an intact form in *E. coli,* and all sequenced plasmids accumulated mutations including point mutations or deletions [26,49]. Whether transformed with the ligation products of PCR amplicons from the *TED* element or isolated plasmids containing *TED*, or directly plated with preserved bacterial solutions of *TED* transformants verified by sequencing, large white colonies occasionally appeared on the LB agar plates (Supplementary Materials Figure S2). Subsequent sequencing of these white colonies unraveled that, except for the linear vector self-ligation products, the insert fragments were *TED*-related sequences bearing different variations, namely, point mutations, solo-terminus deletions or internal deletions, and IS transposon insertions (Figure 4).

First, when we transformed *E. coli* with ligation products of PCR amplicons from the *TED* element, two independent point mutations in *TED* were detected from two biological replicates, respectively (Figure S3). Both were transversion mutations (from guanine to thymine, G1591T and G1606T), resulting in nonsense mutations caused by the presence of an in-frame premature stop codon in the transposase (Figure 4a,b; Supplementary Materials Table S3), whereupon we preferred a hypothesis that cloning hurdles may be related to transposase functions, but this was not the case as shown from subsequent experiments. Solo terminus deletions or internal deletions were also detected among these transformants (Figure 4c–e; Supplementary Materials Table S3), an observation similar to earlier reports of stable mutations caused by the fracture or internal deletions of a transposable element in maize mutable alleles [22,50].

Second, to clarify the genetic stability of the cloned plasmid, both the SNP-borne mutants (G1591T and G1606T) were transformed into *E. coli,* respectively. On LB agar plates, the growth rates of the bacterial colonies transformed with SNP*-TED*-borne plasmids were slightly higher than those transformed with the *TED-*borne plasmid, but much lower than the growth rate of colonies transformed with the *Jittery*-borne plasmid (Figure S4). An internal deletion of 1489-bp was detected from colony PCR and subsequent sequencing analysis of the transformants with the plasmid DNA of the G1591T mutant (Figure 4f). In contrast, three adjacent deletions of vector sequences with varying length (from 284 bp to 963 bp) were detected from three colonies among the transformants with plasmid DNA with the G1606T point mutation (Figure 4g–i; Supplementary Materials Table S3), in addition to the solo-terminus deletions with varied length, which are much more severe mutations compared to the deletions detected from the transformants with straight PCR amplicons of *TED* (Figure 4c,d; Supplementary Materials Table S3). These results indicate that neither *TED* transformants nor their SNP-borne mutants are stable during the normal cycle of *E. coli*.

Third, we focused on the molecular characterization of medium-sized variant colonies to try to unravel their sequence structural basis. There were three internal deletion mutations among them. Two had no trace of sequence micro-homology at the deletion junctions (Figure 4j,k); conversely, one showed a long stretch of micro-homology (*TATCCAGCAG*) at the deletion junction (Figure 4l). These sequences are taken to arise by mechanisms of non-homologous end joining or microhomology-mediated end joining repair of double strand breaks (DSBs). More interestingly, some insertion mutations were frequently detected among the positive transformants. Sequence analysis showed that the exogenous sequences in *TED* were all insertion sequences (IS transposons) from the *E. coli* host (identity over 99%), involving several repeated sequences (IS*3*, IS*4*, and IS*5*) distinguishable by size (Figure 4m–q; Supplementary Materials Table S3). IS*5* was detected in two biological replicates. The two IS*5* sequences were identical, resulting in the deletion of the adjacent *TED* sequence upon insertion (Figure 4p,q). *MULEs* in plant genomes are known to be capable of capturing the host genome sequences in different species [4,5,51,52]. However, in our research, these insertion sequences in *TED* are more likely to be actively inserted from the *E. coli* host into the *TED* in plasmid rather than captured by *TED* from the host genome, since they are all identical to known IS transposons in *E. coli*, and most of them have the standard TSDs flanking the insertions. Similar genetic behavior has been demonstrated in other bacterial genomes [53] and applied to identify active IS and TEs in bacterial plasmid trapping experiments [54,55,56].

To sum up, the solo-terminus deletions occurred more frequently at the 5′-end than at the 3′-end (5:1), whereas internal deletions did not always result from microhomology-mediated end joining repairing, and IS transposon insertions were inserted randomly into the plasmid DNA with respect to their orientation relative to the *TED* element. Furthermore, these de novo mutations from the *TED* transformants suggested that the internal sequence of *TED* (from 1250 to 2845 bp) might be the culprit of the growth defect.

### 2.5. TED Sequence Has No Effect of Inhibition of Bacterial Growth In Vitro

**Figure 5:**
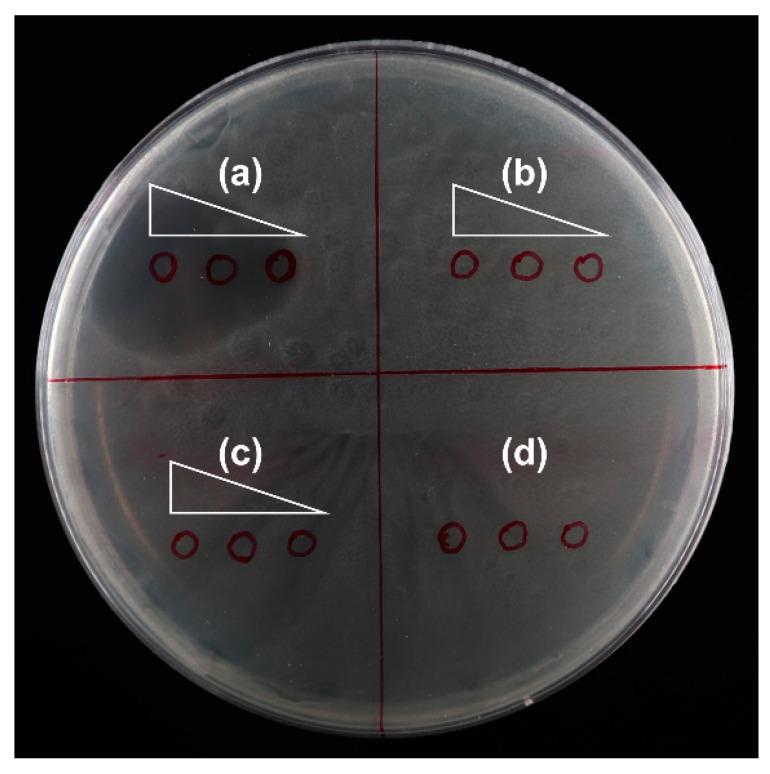
Effects of different solutions on the growth of *E. coli*. (**a**) Kanamycin solution (from left to right, 50 ng/ mL, 5 ng/mL and 0.5 ng/ mL). (**b**) PCR amplicon of *TED* (from left to right, 100 ng/μL, 10 ng/μL and 1 ng/μL). (**c**) Plasmid DNA extraction from *TED* transformants (from left to right, 100 ng/μL, 10 ng/μL and 1 ng/μL). (**d**) Double-distilled water. Red circles are areas applied with testing solutions of various compounds.

The maize *TED* sequence may function as a certain bacteriostatic agent, as seen at a glance, by weakening bacterial vitality, interfering with replication, or destabilizing plasmid multiplication in the process of cloning into an *E. coli* system. To test whether it is practical to apply the *TED* sequence as a bacteriostatic agent for laboratory or industrial purposes, both the *TED* amplicon and plasmid DNA extraction from the *TED* transformants were applied to antibiotic-free LB agar plates with *E. coli* culture for susceptibility test, respectively, where different concentrations of kanamycin were used as the control (Figure 5). The results showed that interpretative microbial responses were observed at various concentrations of kanamycin, resulting in the formation of zones of inhibited growth, and the size of the effectively inhibited areas expanded with the increasement in the antibiotic concentrations (Section a). Nevertheless, across a broad range of concentrations, neither the *TED* nucleic acid solution nor plasmid solution of the *TED* transformants inhibited the growth of *E. coli* (Sections b and c), in line with the results from double-distilled water, negative controls (Section d). These in vitro studies show that either the *TED* nucleic acid solution or plasmid solution of the *TED* transformants has a bacteriostatic effect. The hampered bacterial growth during the transformation process was most likely due to the *TED* transformed plasmid in *E. coli*, but not the in vitro solution used for transformation.

### 2.6. Complex Sequence Structure Formed by TED May Be the Hurdle for Cloning

**Figure 6:**
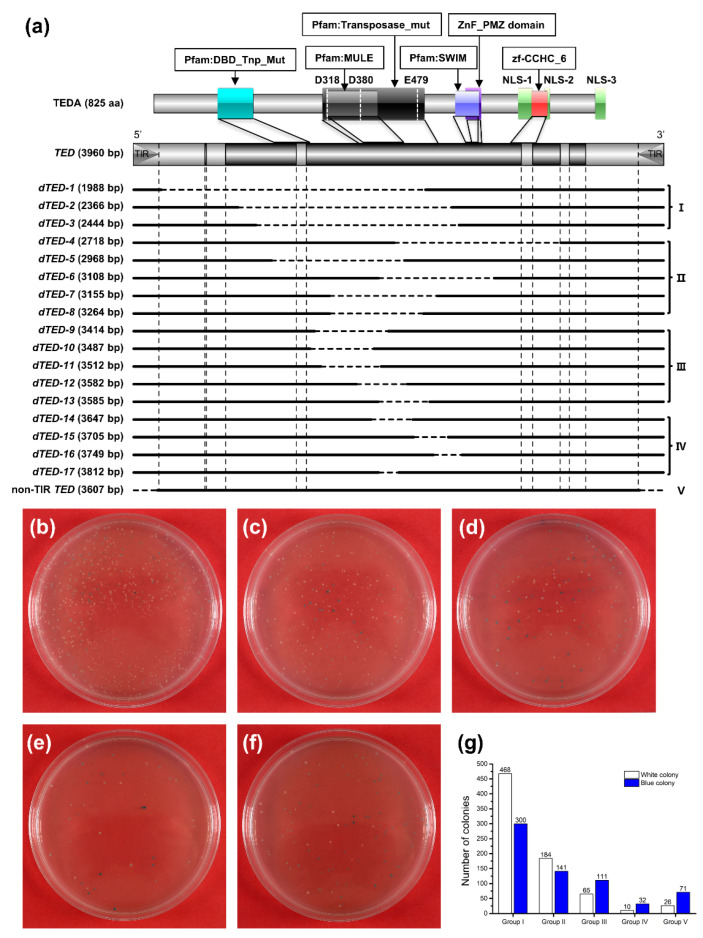
Schematic representation of *dTED* used for cloning in *E. coli* and the growth dynamics of *dTED* groups with various sizes by the modified method. (**a**) Defective *TED* used for cloning. The top part is TEDA with the conserved domains and the *TED* full-length genomic DNA with the gene structure. Deletions are shown as the horizontal dotted lines. TIRs and exon–intron junctions are indicated by the vertical dotted lines. The plating culture growth of the PCR amplicons were examined after 36 h of incubation at 37 °C. (**b**–**e**) *dTED* group I to IV. (**f**) Non-TIR *TED,* group V. (**g**) The counts of different colonies from the corresponding plating culture of different cloning inserts (**a**–**e**).

Apparently, some sequence composition shared between the intact genomic copy of the *TED* element and its full-length cDNA is the hurdle that accounts for the retarded growth of the bacterial host, their slacken multiplication, and highly mutable replication of plasmid transformants with intact *TED* or their SNP variants. To identify the causative sequences, 17 previously isolated *dTED* elements, resulting from internal deletions of the coding region of the functional TEDA transposase, were amplified for subsequent cloning into *E. coli* via the modified method. Meanwhile, a non-TIR *TED* fragment was also amplified for cloning to clarify whether or not TIR sequences are involved in the cloning hurdles. These PCR products were divided lengthwise into five groups for an efficient cloning trial (Figure 6a and Supplementary Materials Table S4). Under an optimized experimental condition as described before, all *dTED* sequences were successfully cloned (Figure 6b–f), and the number of positive colonies was higher when the length of the cloned inserts was smaller. Although the PCR amplified non-TIR *TED* fragment could be cloned in *E. coli*, the number of colonies was fewer and the colony size was tiny in the colonies from intact *TED* transformants, suggesting that TIRs are not the “toxic” zones in the *TED* element.

Of the larger-sized *dTED* amplicons, all positive clones were identified from tiny colonies resulting from the retarded growth, so these *dTED* elements may bear toxic sequences, leading to cloning hurdles in spite of their coding for nonfunctional transposase. It is well-known that some genes cannot be cloned with conventional protocols because, in most cases, these genes or their products are toxic to *E. coli* such as membrane protein genes [57,58], enzyme genes [59,60,61], and flavivirus members [62,63,64,65,66] because of various harmful effects to the *E. coli* host. The molecular cloning of these genes has been a major challenge; consequently, direct studies of their activities in heterologous expression systems remain impossible.

So far, the leaky expression of functional transposase would not be the cause for the failure in cloning *TED* into *E. coli* by standard molecular biological techniques, since no leaky expression of functional transposase would possibly occur in the *dTED* transformants. Therefore, the cloning abnormalities encountered result from other novel mechanism(s).

**Figure 7:**
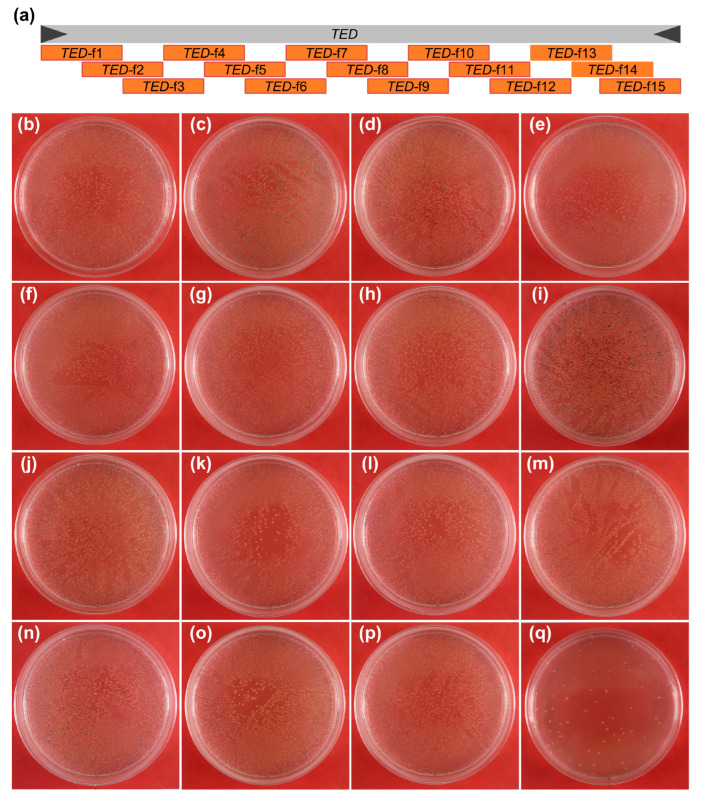
Cloning of the fragmented *TED* element. (**a**) Schematic representation of the fragmentation of *TED* for cloning to screen for toxic sequences to *E. coli*. The gray rectangle is the *TED* nucleic acid sequence, the dark gray triangles are the TIRs of *TED*. The orange rectangles are fragments of *TED* amplified by PCR and used for cloning in *E. coli.* (**b**–**q**) Growth dynamics of the colonies transformed with different *TED* fragments and the intact *TED* sequence. All sequences were cloned by the modified cloning method. (**b**–**p**) *TED* Fragment-1 to *TED* Fragment-15 shown in (**a**). (**q**) Intact *TED* sequence.

To further explore which regions of the *TED* sequence affected the vitality of the colonies, the entire *TED* sequence was divided into 15 fragments of 500-bp in length. Fragments next to each other shared 250-bp overlap sequences with upstream and downstream fragments, except for the fragment-15, which was a 500-bp sequence at the 3′-end of *TED* (Figure 7a). All 15 fragments were PCR amplified with corresponding PCR primer combinations and cloned into *E. coli* when *TED* was used as the experimental control. Thousands of positive colonies were unexpectedly obtained from all transformations of the 15 fragments (Figure 7a–p), while only dozens of white colonies were obtained from transformation with *TED* (Figure 7q). It is deducible that the mysterious hurdle for cloning the *TED* transposon was not caused by any certain segment of the *TED* element alone. We further speculate that the long nucleotide sequences such as the intact *TED* element, its SNP mutations, or full-length cDNA possess multiple minor-effects or toxic motifs interacted with each other to interfere with the DNA replication or bacteria proliferation due to the structure complex formed in the host bacteria cell.

## 3. Discussion

*MuDR*, the autonomous DNA transposon first described in maize, is the founding member of the *Mutator* superfamily [7]. For decades, our knowledge of the *Mutator* superfamily remained largely confined to *MuDR* and enormous non-autonomous *Mu-like* elements in the field of their regulation, evolution, and the practical application in functional genomics [49]. *TED* and *Jittery* are two recently characterized autonomous transposons of the *Mutator* superfamily in maize [21,22]. Despite numerous conserved features among these three elements, both *TED* and *Jittery* have unique sequence structures and display genetic behaviors distinct from *MuDR* [47]. The full-length cDNA of both elements were cloned using 3′ and 5′ RACE-PCR. Subsequent sequence analysis showed that TEDA transposase shared all conserved domains and NLSs with MURA, while JITA was distantly related to MURA, which conclusively proved the previous predictions [21,22,49].

Standard *MuDR* elements from different *Mutator* lines of maize and genetically active *MuDR*-like elements from some accessions of the Mexican land race Zapalote chico are toxic to *E. coli*, so they must be cloned as overlapping fragments [26,32,34]. Due to the fact that it is difficult to be cloned in *E. coli*, the biochemistry of MURA activity is limitedly understood, and the application of the *MuDR/Mu* system is confined to maize. In our study, we found that *TED*, *Jittery*, and their full-length cDNA sequences are difficult to clone in *E. coli* with the standard protocols from several manufacturers, *TED* and its cDNA being the hardest. Therefore, the refractoriness of cloning is not confined to *MuDR* and the result further validates the earlier predictions about the relationship between these three elements [49].

Several labs have failed to maintain the intact *MuDR* or *mudrA*, even using recombination-defective *E. coli* strains [26,34]. The troublesome analysis of the MURA protein function was overcome with the stabilization of *mudrA* cDNA in a yeast plasmid and the successful expression of MURA from a yeast-inducible promoter construct. The expression of MURA in a heterologous host initiated the functional characterization of MURA, particularly with respect to its DNA-binding properties [34]. By appropriately extending the bacterial recovery time prior to plating, we successfully cloned the *TED* element and its full-length cDNA in *E. coli*. However, the growth of positive colonies bearing *TED* or its cDNA transformants was greatly retarded. We thought that the DNA replication or gene expression in the bacterial cell may be heavily inhibited. When extending the time for incubation, the bacteria had a longer time to recover from heat shock, which favors the survival of bacteria transformed with *TED* or its cDNA on the selective LB plates. However, a much longer incubation time would result in the growth of various mutated colonies on the LB plates. It suggests that the mutated colonies have gone through generations of proliferation in LB broth prior to plating, a similar process leading to the recovery of normal sized colonies during the cloning of *TED* or its cDNA transformants. Furthermore, we serendipitously discovered the polychromatic sequence landscape of *TED* transposons surviving from arduous growth in *E. coli*. In addition to the multifarious variants reported earlier, multiple sequence rearrangements resulting from bacterial IS transposon insertions were also detected, which further supports the mechanism of IS transposition mediated genetic information transfer to bacterial plasmids [67]. Because double-strand breaks generated by IS transposition might induce error-prone repair, the deletion detected in the *TED* sequence may also be caused by IS elements.

Previous research suggests that the Shine–Dalgarno motif may cause *MuDR* instability as it allows for transposase production in *E. coli* [36]. However, neither the *TED* full-length genomic DNA nor cDNA contained Shine–Dalgarno-motif-like sequences. Indeed, both fragments are difficult to clone in *E. coli*. Therefore, the Shine–Dalgarno motif might be a unique cause to the cloning of the refractory *MuDR* element, and it is likely that sequence features other than the Shine–Dalgarno motif from *MuDR* have evolved since the separation of the *MuDR* and *TED* clades. Although the terminal inverted repeat sequence in plasmid can fold in perfectly paired stable secondary structures that would be frequently deleted in *E. coli* [68], our results indicated that TIRs at both ends of *TED* were not the primary cause of refractory cloning. In addition, we ruled out the effects of leaky expression of TEDA transposase on cloning because neither the non-TIR *TED* nor *TED* cDNA could be cloned by the standard method. In fact, certain sequences cannot be cloned by conventional methods due to different reasons. For example, sequences containing numerous tandem or inverted repeats can lead the circular plasmid to generate secondary structures that are substrates for deletion, which makes sequences unstable in *E. coli* [69]. Furthermore, the leaky expression of recombinant protein in bacteria can also impede cloning in *E. coli* due to the inhibitory effect of protein products on cell proliferation [57,59,62]. These results indicated that it is feasible to ligate the plasmid vector with *TED* and *TED-*related sequences efficiently and to transform normally prior to the direct take up of the plasmid by bacterial cells. In addition, an internal 1595 bp segment (from 1250 bp to 2845 bp in *TED* genomic sequence) is likely to be the culprit of the growth defect. It is worthwhile identifying a certain specific structure or sequence composition in DNA fragments such as hairpins, triplexes, slipped structures, or highly flexible and writhed helices, which may impair bacterial activity and cause instability [69]. Apparently, further essential research may focus on the understanding of how *MuDR* and *TED* affect bacterial metabolism and the expression of genes involved in DNA replication and the formation of nucleoprotein complexes, which are crucial to mediate bacterial genome organization [70] and their evolutionary significance.

## 4. Materials and Methods

### 4.1. Genetic Stocks

All *TED* stocks (*bz-m175::dTED; trTED*), originally from the Dooner lab, were maintained in the Li lab. An intact *Jittery* element was extracted from an *Mrh* stock obtained from the Maize Genetics Cooperation Stock Center. Interestingly, this element can *trans*-activate the transposition of the *rMrh* element in the *a1-rMrh* allele (Yubin Li, unpublished observation).

### 4.2. Preparation of RNA and Rapid Amplification of cDNA Ends

The total RNA was extracted from the maize seedling leaves by a modified TRIzol-based method with the Transzol reagent (TransGen Biotech, Beijing, China) and the DNA was degraded by DNase I (GenStar, Beijing, China) following the manufacturer’s protocol. The 5′- and 3′-ends of the cDNA were cloned by the SMARTer^®^ RACE 5′/3′ Kit (Takara, Dalian, China) following the user manual and fused through overlap PCR. The gene-specific primers used in RACE are listed in Supplementary Materials Table S1.

### 4.3. Protein Sequence Analysis

Full-length cDNA sequences were translated in silico into protein sequences for bioinformatics functional analysis. The basic physical and chemical properties of the proteins were determined using the ProtParam tool (https://web.expasy.org/protparam/, accessed on 10 September 2021). Conserved domains and motifs were detected through a combination of SMART (Simple Modular Architecture Research Tool, website, http://smart.embl.de, accessed on 10 September 2021) and the MOTIF search tool (GenomeNet, https://www.genome.jp/tools/motif/, accessed on 10 September 2021). Nuclear localization sequences were predicted using the NLStradamus website (http://www.moseslab.csb.utoronto.ca/NLStradamus/, accessed on 10 September 2021). To clarify the DDE triad and other conserved amino acids of the transposase, a multiple sequence alignment of the predicted proteins (MURA, TEDA and JITA) was performed using the COBALT tool on the NCBI website (http://www.ncbi.nlm.nih.gov/tools/cobalt/, accessed on 17 October 2021) and output with DNAMAN software. Finally, the schematics of the gene structure and protein composition were produced using IBS (Illustrator for Biological Sequences) software [71].

### 4.4. Modification of Cloning Protocols and Inserts Identification

All amplicons were obtained by nested PCR with the high-fidelity enzyme (Vazyme, Nanjing, China) and purified with the DNA Gel Extraction Kit (Axygen, Hangzhou, China) prior to cloning. The purified PCR products were ligated by the Blunt End Cloning Kit (TransGen Biotech, Beijing, China) according to the recommended reaction conditions. The ligation products were then transformed into competent cells of *E. coli Trans*1-T1 (TransGen Biotech, Beijing, China), derived from the original strain of K12. The incubation time after heat shock was extended to 70 min without changing the other experimental steps. A total of 200 μL of competent cell recovery solution was coated on Luria-Bertani (LB) solid medium supplemented with kanamycin, IPTG, and X-gal. When incubated at 37 °C, the colony growth was photographed every 12 h. Colony PCR was performed to identify cloning inserts from all tiny, small, and large colonies with the poly-linker primer of M13F and M13R.

## Data Availability

Not applicable.

## Supplementary Materials

The supporting information can be downloaded at: https://www.mdpi.com/article/10.3390/ijms231911993/s1.
