# Supplementary material for: Cloning of Maize TED Transposon into Escherichia coli Reveals the Polychromatic Sequence Landscape of Refractorily Propagated Plasmids
Source: Int J Mol Sci. 2022 Oct 9;23(19):11993. doi: 10.3390/ijms231911993 (PMC9569675; doi:10.3390/ijms231911993)
Supplement: Supplementary file 1 [file ijms-23-11993-s001.zip › Figure S1.pptx]

## Slide 1
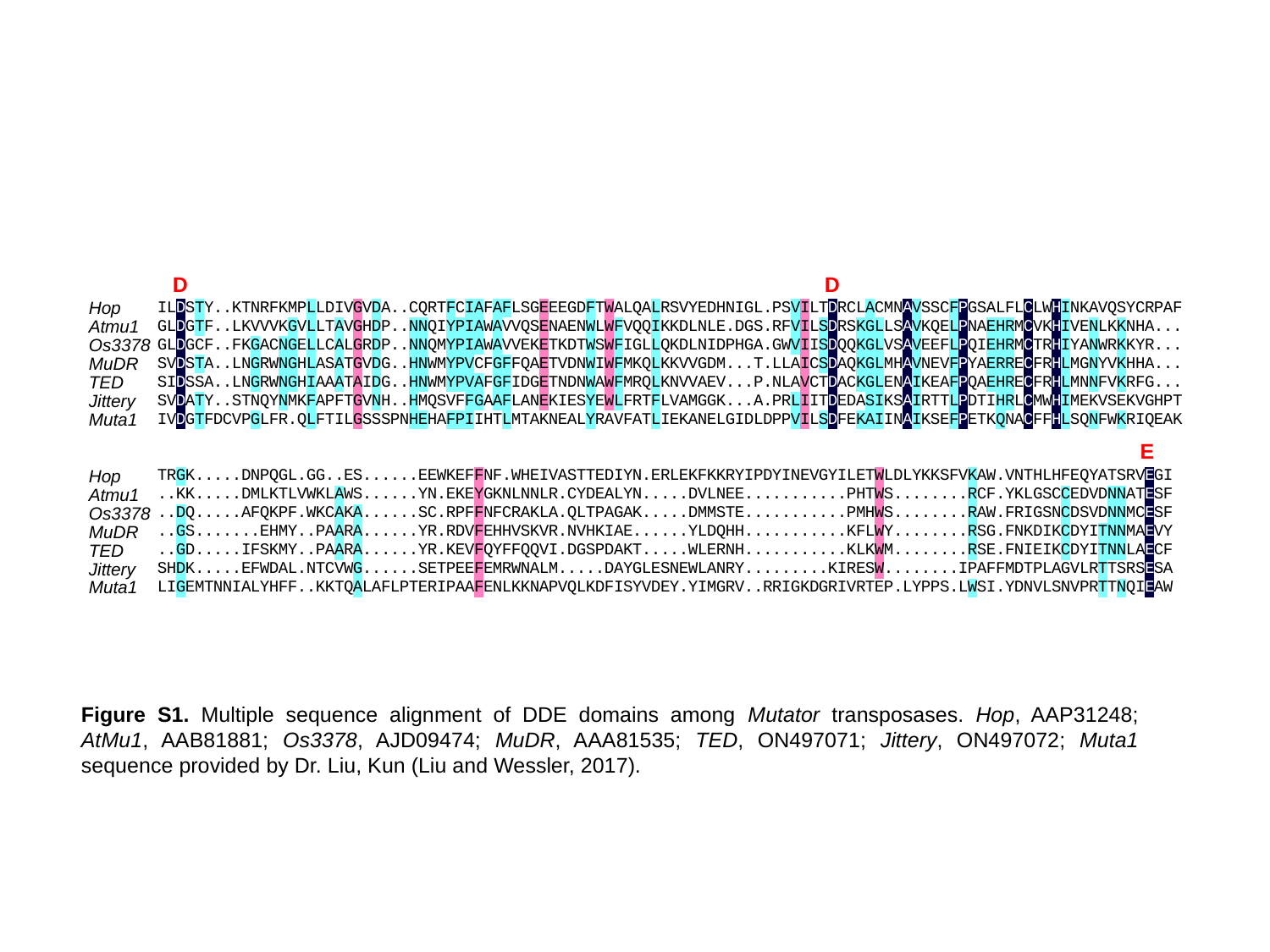

D
D
E
Hop
Atmu1
Os3378
MuDR
TED
Jittery
Muta1
Hop
Atmu1
Os3378
MuDR
TED
Jittery
Muta1
Figure S1. Multiple sequence alignment of DDE domains among Mutator transposases. Hop, AAP31248; AtMu1, AAB81881; Os3378, AJD09474; MuDR, AAA81535; TED, ON497071; Jittery, ON497072; Muta1 sequence provided by Dr. Liu, Kun (Liu and Wessler, 2017).
