# Supplementary material for: Cloning of Maize TED Transposon into Escherichia coli Reveals the Polychromatic Sequence Landscape of Refractorily Propagated Plasmids
Source: Int J Mol Sci. 2022 Oct 9;23(19):11993. doi: 10.3390/ijms231911993 (PMC9569675; doi:10.3390/ijms231911993)
Supplement: Supplementary file 1 [file ijms-23-11993-s001.zip › Figure S2.pptx]

## Slide 1
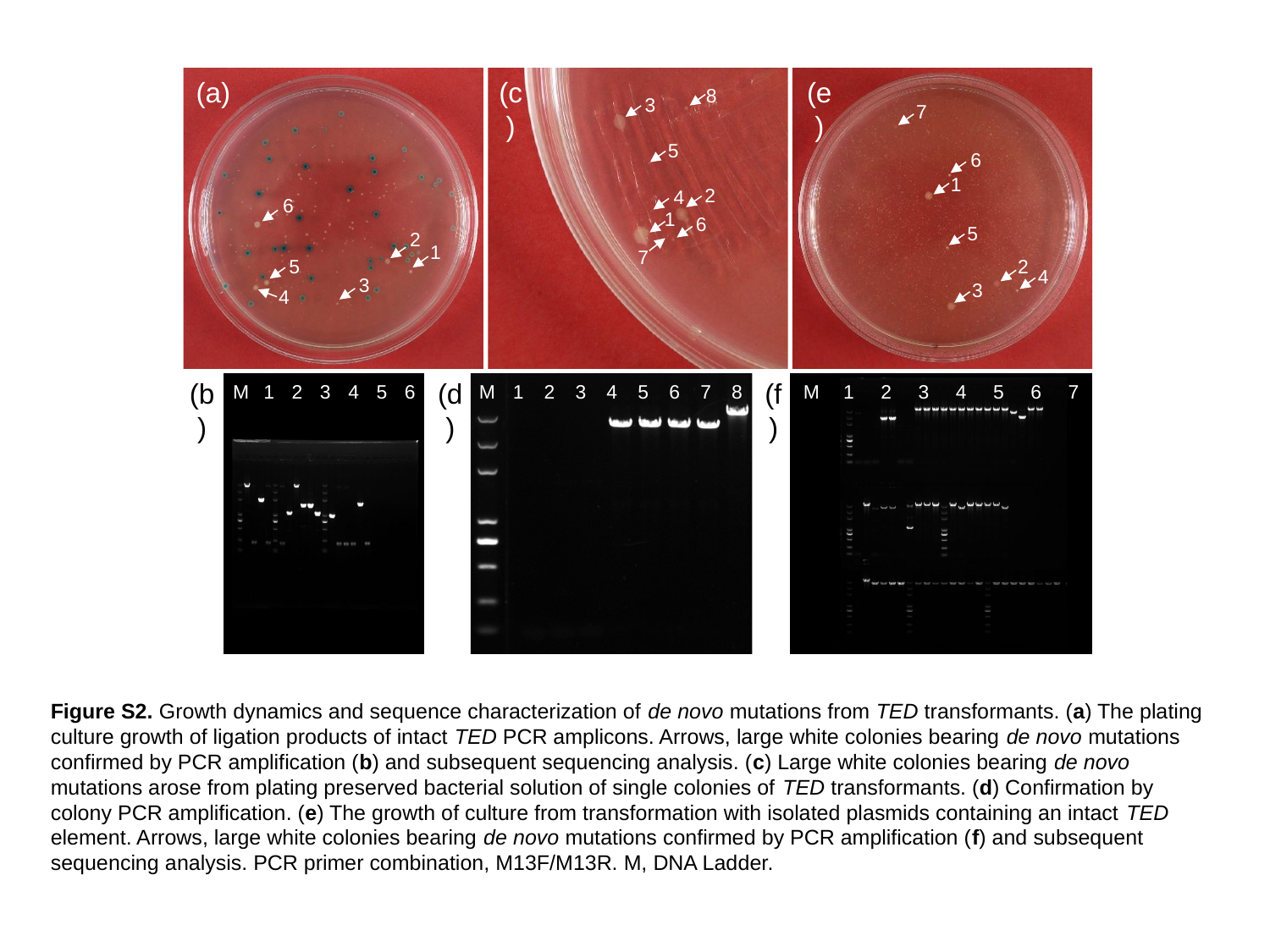

(a)
6
2
1
5
3
4
(c)
8
3
5
2
4
1
6
7
(e)
7
6
1
5
2
4
3
(b)
(d)
(f)
M
1
2
3
4
5
6
7
8
M
1
2
3
4
5
6
7
M
1
2
3
4
5
6
M
1
2
3
4
5
6
7
8
Figure S2. Growth dynamics and sequence characterization of de novo mutations from TED transformants. (a) The plating culture growth of ligation products of intact TED PCR amplicons. Arrows, large white colonies bearing de novo mutations confirmed by PCR amplification (b) and subsequent sequencing analysis. (c) Large white colonies bearing de novo mutations arose from plating preserved bacterial solution of single colonies of TED transformants. (d) Confirmation by colony PCR amplification. (e) The growth of culture from transformation with isolated plasmids containing an intact TED element. Arrows, large white colonies bearing de novo mutations confirmed by PCR amplification (f) and subsequent sequencing analysis. PCR primer combination, M13F/M13R. M, DNA Ladder.
