# Supplementary material for: Cloning of Maize TED Transposon into Escherichia coli Reveals the Polychromatic Sequence Landscape of Refractorily Propagated Plasmids
Source: Int J Mol Sci. 2022 Oct 9;23(19):11993. doi: 10.3390/ijms231911993 (PMC9569675; doi:10.3390/ijms231911993)
Supplement: Supplementary file 1 [file ijms-23-11993-s001.zip › Figure S3.pptx]

## Slide 1
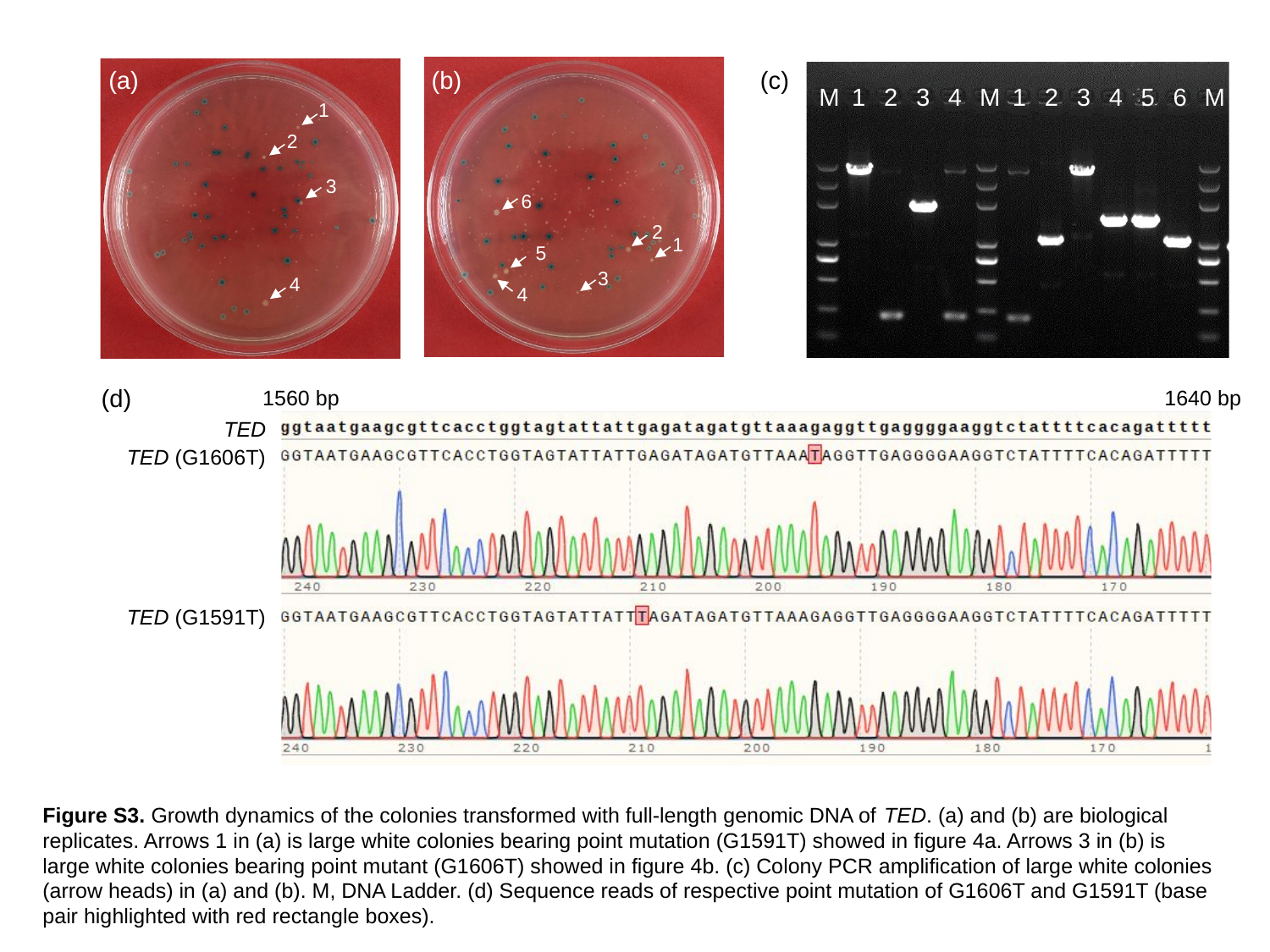

6
2
1
5
3
4
(a)
(b)
(c)
M
1
2
3
4
M
1
2
3
4
5
6
M
1
2
3
4
(d)
1560 bp
1640 bp
TED
TED (G1606T)
TED (G1591T)
Figure S3. Growth dynamics of the colonies transformed with full-length genomic DNA of TED. (a) and (b) are biological replicates. Arrows 1 in (a) is large white colonies bearing point mutation (G1591T) showed in figure 4a. Arrows 3 in (b) is large white colonies bearing point mutant (G1606T) showed in figure 4b. (c) Colony PCR amplification of large white colonies (arrow heads) in (a) and (b). M, DNA Ladder. (d) Sequence reads of respective point mutation of G1606T and G1591T (base pair highlighted with red rectangle boxes).
