# Supplementary material for: Cloning of Maize TED Transposon into Escherichia coli Reveals the Polychromatic Sequence Landscape of Refractorily Propagated Plasmids
Source: Int J Mol Sci. 2022 Oct 9;23(19):11993. doi: 10.3390/ijms231911993 (PMC9569675; doi:10.3390/ijms231911993)
Supplement: Supplementary file 1 [file ijms-23-11993-s001.zip › Figure S4.pptx]

## Slide 1
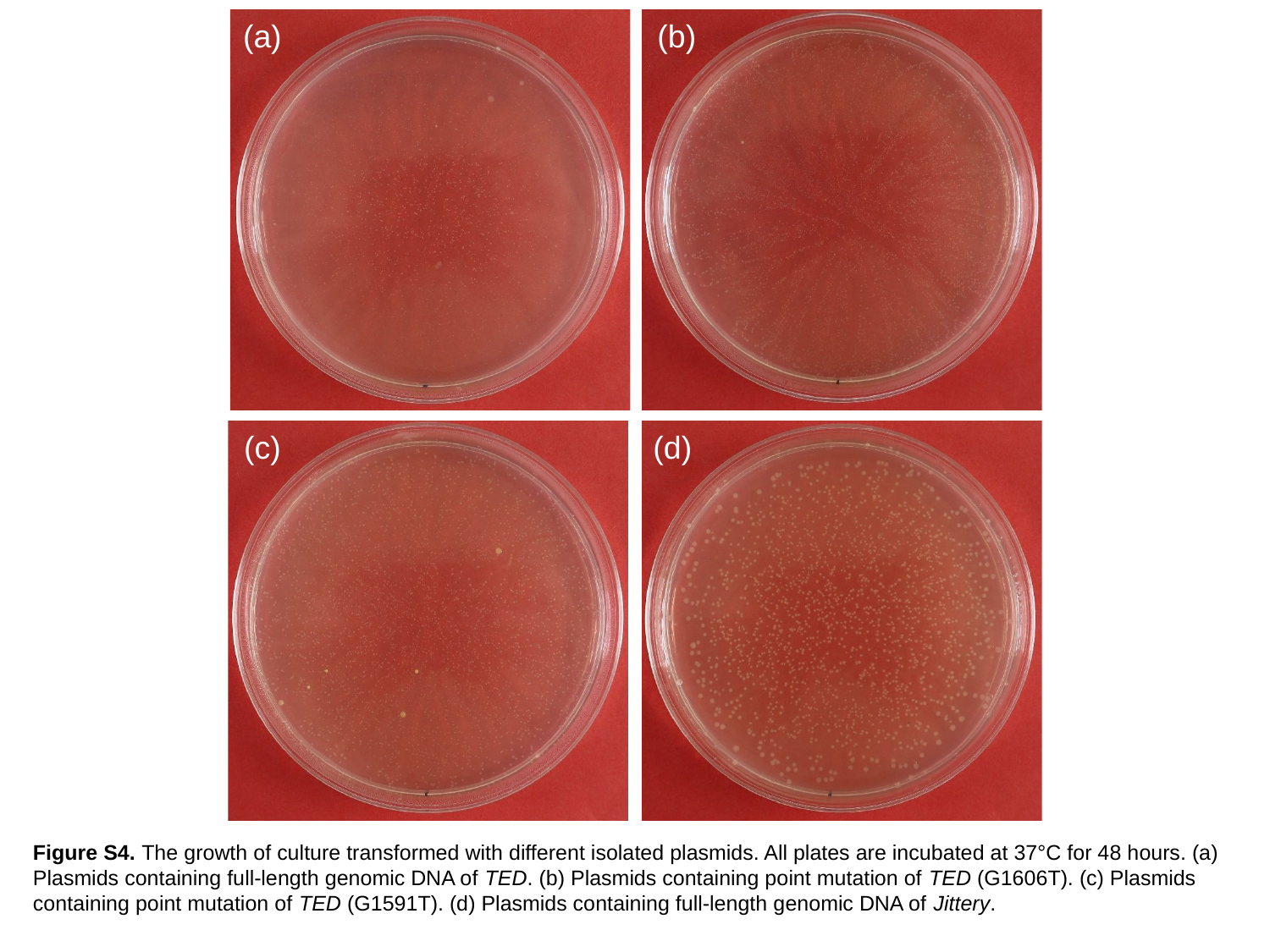

(a)
(b)
(c)
(d)
Figure S4. The growth of culture transformed with different isolated plasmids. All plates are incubated at 37°C for 48 hours. (a) Plasmids containing full-length genomic DNA of TED. (b) Plasmids containing point mutation of TED (G1606T). (c) Plasmids containing point mutation of TED (G1591T). (d) Plasmids containing full-length genomic DNA of Jittery.
